# Sudden sensorineural hearing loss in COVID‐19: A case report and literature review

**DOI:** 10.1002/ccr3.4019

**Published:** 2021-03-11

**Authors:** Eline Beckers, Pascale Chouvel, Valérie Cassetto, Vincent Mustin

**Affiliations:** ^1^ Department of Otorhinolaryngology‐Head and Neck Surgery Cliniques de l’Europe Brussels Belgium; ^2^ Department of Radiology Cliniques de l’Europe Brussels Belgium

**Keywords:** coronavirus, COVID‐19, deafness, SARS‐CoV‐2, sudden sensorineural hearing loss

## Abstract

We encourage SARS‐CoV‐2 polymerase chain reaction in the diagnostic workup of patients currently presenting with sudden sensorineural hearing loss.

## Graphical abstract

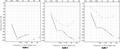

## 1 INTRODUCTION

Since the beginning of the COVID‐19 pandemic, the clinical profile of patients with SARS‐CoV‐2 infection has been extensively investigated, also in the otorhinolaryngological field. The virus was found to trigger anosmia in a substantial number of infected patients,^1^ and furthermore, a link with sudden sensorineural hearing loss (SSNHL) has been suggested.

A first note on the link between COVID‐19 and hearing loss was published online in April 2020 by Sriwijitalai et al^2^ In June 2020, Karimi‐Galougahi et al reported three cases of SSNHL in patients with positive SARS‐CoV‐2 polymerase chain reaction (PCR), raising the need to closely examine this new association.^3^

Our PubMed search, conducted in November 2020 with the search terms “COVID”, “Corona”, “Coronavirus”, “hearing loss” and “deafness”, retrieved only 6 reports consisting of 8 audiometrically confirmed cases of SSNHL in SARS‐CoV‐2‐infected patients worldwide.^3^, ^4^, ^5^, ^6^, ^7^, ^8^ The cases are summarized in Table 1.

**TABLE 1:** Summary of the existing case reports on sudden sensorineural hearing loss in SARS‐CoV‐2‐positive patients

N	Article (2020)	Author (reference)	Sex	Age (years)	Laterality of SSNHL	4‐frequency PTA (dB)	Treatment	Outcome after reported FU
1	May	Karimi (3)	M	22	Left	0 ‐ 0 – 30 ‐ 40	Unknown	Unknown
2	May	Karimi (3)	F	40	Right	50 ‐ 60 ‐ 60 ‐ 60	Unknown	Unknown
3	May	Karimi (3)	F	23	Left	30 ‐ 35 ‐ 35 ‐ 40	Unknown	Unknown
4	June	Degen (4)	M	60	Bilateral	Unknown	Urgent cochlear implantation (Right), 3 IT injections (Left)	Unknown
5	June	Kilic (5)	M	29	Right	50 ‐ 40 ‐ 20 ‐ 10	Hydroxychloroquine	Complete hearing recovery
6	September	Lang (6)	F	30	Right	15 ‐ 10 ‐ 95 ‐ 80	Oral prednisolone	No significant improvement
7	September	Koumpa (7)	M	45	Left	Unknown	Oral prednisolone and 3 IT injections	Partial improvement
8	September	Lamounier (8)	F	67	Right	60 ‐ 85 ‐ 75 ‐ 75	Oral prednisolone and 5 IT injections	Isolated recovery at 250Hz

Note

The audiometric data of the hearing loss at onset are reported as 4‐frequency pure‐tone average at respectively 500Hz ‐ 1000Hz ‐ 2000Hz ‐ 3000 or 4000Hz frequencies.

Abbreviations: dB, Decibels; F, Female; FU, Follow‐up period; Hz, Hertz; IT, Intratympanic corticoid; M, Male; N, Number; PTA, pure‐tone average; SNHL, Sensorineural hearing loss.

### 1.1 Case description

**FIGURE 1:**
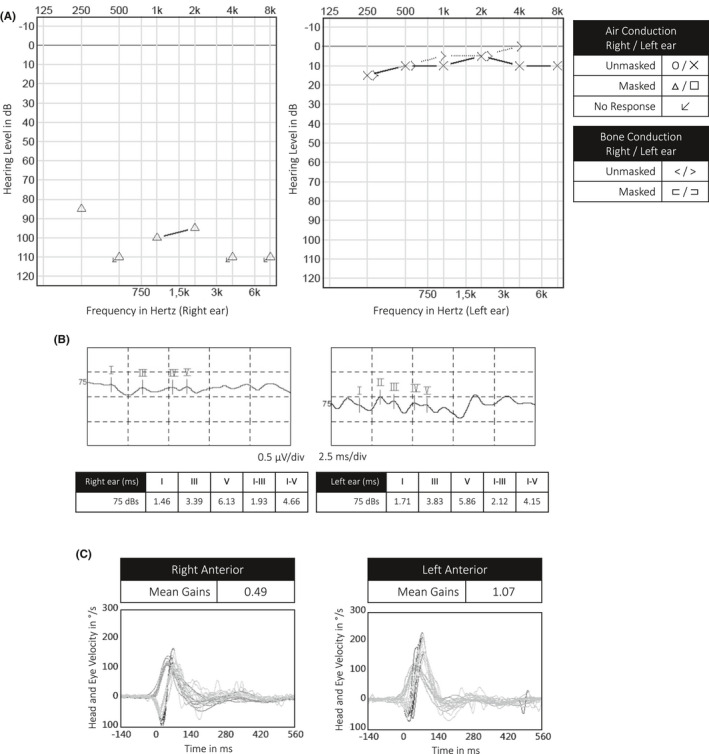
Audiometric and vestibular findings at onset of the sudden sensorineural hearing loss in our case (referred to as “Audio 1” in Figure 3). A: Tonal audiometry. B: Brainstem Auditory Evoked Potentials. C: Video Head Impulse Testing

We present the 9th case of SSNHL in a SARS‐CoV‐2‐positive patient. Written informed consent was obtained. This patient, a 53‐year‐old man of Turkish origin, presented at the emergency department in the beginning of November 2020 because of a sudden right‐sided hearing loss. He mentioned fatigue and a subfebrile status in the past twelve days. Anamnestically, there were no other general nor neuro‐otological symptoms such as vertigo or instability. The patient had no medical history and was not taking any medication. On the day of referral to the emergency department, a SARS‐CoV‐2 PCR was performed by his general practitioner and proved to be positive. Clinical and technical examination by an otorhinolaryngologist showed normal otomicroscopy, normal tympanometry, and a cophosis on the right side. Video Head Impulse Testing showed a deficit of the right anterior semicircular canal. Audiometric and vestibular findings are shown in Figure 1A‐C. Blood analysis showed normal hematologic and serum biochemical levels, except for an elevated fibrinogen (6.9 g/L, range 2.0‐4.0 g/L), and CRP (84 mg/L, range < 10 mg/L). Serology was performed, showing presence of specific Epstein‐Barr virus and Cytomegalovirus IgG in absence of specific IgM.

**FIGURE 2:**
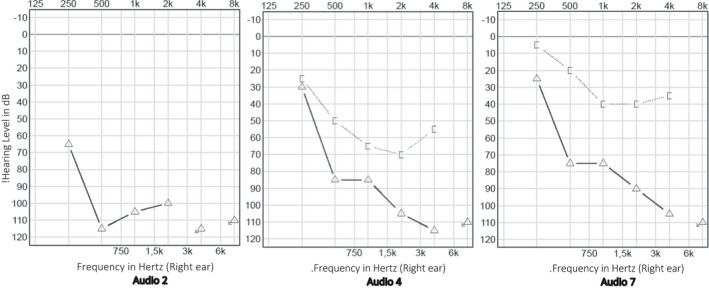
Tonal audiometry at three follow‐up evaluations (referred to as “Audio 2, 4, and 7,” respectively, in Figure 3)

**FIGURE 3:**
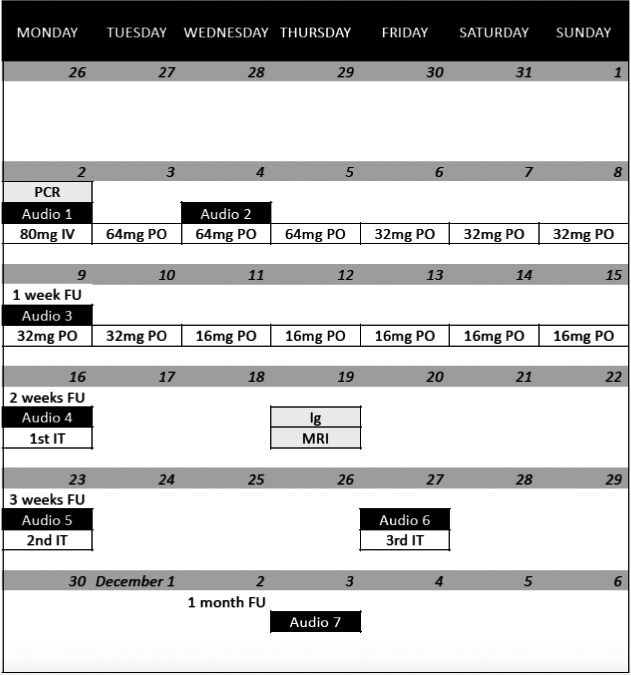
Timing of diagnostic assessments and therapeutical interventions during the first month of follow‐up of our case (November 2020). Abbreviations: Intravenous corticosteroid treatment (IV), Per oral corticosteroid treatment (PO), Intratympanic corticosteroid treatment (IT), Audiometric evaluations 1‐7 (Audio 1‐7), Analysis of SARS‐CoV‐2 polymerase chain reaction (PCR), Analysis of SARS‐CoV‐2 IgG/IgM (Ig), Magnetic resonance imaging of the temporal bones (MRI), Follow‐up period (FU)

A single dose of methylprednisolone 80 mg and piracetam 12 g was administered intravenously. After shared decision‐making, the patient was discharged from the hospital with oral treatment consisting of methylprednisolone once daily, piracetam 2.4 g twice daily, zinc, and a proton‐pump inhibitor. Regular follow‐up consultations were planned. Tonal audiometry thresholds successively improved for each evaluation, but only in the lower and middle frequencies (Figure 2). The patient did not mention a subjective hearing improvement until two weeks after onset of SSNHL. The per oral corticosteroid treatment was followed by right‐sided intratympanic injections of 2 mL dexamethasone (5 mg/mL). The patient underwent magnetic resonance imaging of the temporal bones, excluding retrocochlear pathology. Serology performed after two weeks showed presence of SARS‐CoV‐2 antibodies (IgM + IgG total of 15.8 UA/mL, range < 1.0 UA/mL, no IgM/IgG subdivision known). One month after onset of SSNHL, a disabling unilateral hearing loss remained. The timing of diagnostic assessments and therapeutical interventions is visualized in Figure 3.

## 2 DISCUSSION

SSNHL is mostly defined as a sensorineural hearing loss of at least 30 decibels (dB) at three consecutive frequencies, occurring within a timeframe of 72 hours. It can affect any age group, but mostly occurs in patients aged 40‐60 years. In the majority of cases, adequate investigation does not reveal any underlying cause (eg, vestibular schwannoma, stroke, malignancy, and exposure to noise or ototoxic medication), so that the SSNHL is considered as idiopathic.^9^, ^10^ However, multiple etiologies have been proposed.

First, SSNHL has often been associated to viral infection. Serological techniques such as PCR and immunoglobulin detection have previously been used to confirm viral load in patients with SSNHL. However, a direct causality between acute viral infection and SSNHL remains uncertain since in vivo sampling of inner ear fluid is potentially harmful. Second, vascular impairment is suggested as a cause of SSNHL. Both cardiovascular risk factors and virally induced hypercoagulability or inflammatory edema can theoretically lead to cochlear ischemia with subsequent hearing loss.^10^

Thus, how about the specific SARS‐CoV‐2 infection and occurrence of hearing loss? Interestingly, the audiological profile of patients with asymptomatic SARS‐CoV‐2 infection was examined, showing significantly worse high‐frequency pure‐tone threshold amplitudes and transient evoked oto‐acoustic emissions in these patients compared with noninfected subjects. This finding suggests a damaging effect on cochlear outer hair cell function.^11^

It is known that SARS‐CoV‐2 affects cells through the angiotensin‐converting enzyme 2 (ACE2) receptor. This receptor is expressed in various cell types, such as lung respiratory epithelium and the sustentacular cells of the olfactory neuroepithelium. On the one hand, viral infection of the auditory nervous system could induce symptoms in a similar way as infection of the olfactory nervous system is believed to contribute to the pathophysiology of COVID‐anosmia.^1^, ^12^ On the other hand, coagulopathy with high incidence of thrombotic events is well‐described in COVID‐19 patients.^13^ ACE2 receptors are expressed in endothelial cells in various organs as well. The important findings of Varga et al taught us that SARS‐CoV‐2 promotes the induction of endotheliitis. The team detected SARS‐CoV‐2 elements in endothelial cells, with evidence for the induction of endothelial dysfunction and cell apoptosis.^14^ Hence, as hypothesized by Harenberg et al, hearing loss in COVID‐19 patients could be a result of endothelial cell dysfunction with micro‐thrombosis at the level of the auditory center in the temporal lobe, the auditory nerve or the cochlea.^12^ In the case of Degen et al however, the sudden hearing loss is described as a neurological complication of viral meningitis.^4^

COVID‐19 is postulated to be the cause of SSNHL in our case. The risk of a false association, in particular because of the unclarified pathophysiology of SSNHL, should however be stated. Therefore, we agree with and applied the criteria of Satar et al before proposing the relationship between SARS‐CoV‐2 infection and occurrence of SSNHL in our case.^15^ First, the infection was laboratory‐confirmed. Second, pathological ipsilateral Video Head Impulse Testing assigns for vestibular involvement, although our patient did not complain of associated symptoms. Next, other causes of SSNHL have been excluded anamnestically, serologically and by magnetic resonance imaging. Last, the temporal concordance between SARS‐CoV‐2 infection and onset of SSNHL remains unresolved. Four out of the nine cases did not report the onset of SSNHL. All five cases that did mention the onset of hearing loss, describe occurrence between at least 12 and 47 days after the first COVID‐19 symptoms. We could therefore hypothesize that SSNHL in SARS‐CoV‐2 infection is more likely to appear in the downward phase of the infection; however, three patients were only able to notice their hearing loss when released from the intensive care unit.

## 3 CONCLUSION

This case report adds new well‐founded evidence to the association between COVID‐19 and hearing loss. We hope it will contribute to the prompt recognition of both SSNHL and SARS‐CoV‐2 infection. Based on this case and literature review, we advise to include PCR testing in the diagnostic workup of patients presenting with SSNHL during the ongoing pandemic. Whether the sudden hearing loss is the result of endothelial cell dysfunction with micro‐thrombosis in the cochlea or central auditory pathways remains unclear.

## CONFLICT OF INTEREST

None declared.

## AUTHOR CONTRIBUTIONS

BE wrote the manuscript. CP contributed to conceptualizing, writing, and reviewing. CV contributed to radiological management of the patient. MV contributed to reviewing and supervision.

## ETHICS STATEMENT

This case report was written and published with consent of the patient.

## Data Availability

All data are available from the corresponding author upon request.
